# Kasabach-Merritt Syndrome in an Adult: A Comment

**DOI:** 10.4274/tjh.galenos.2018.2018.0356

**Published:** 2019-02-07

**Authors:** Sevgi Gözdaşoğlu

**Affiliations:** 1 Retired Professor

**Keywords:** Megadose methylprednisone, Kasabach-Merritt Syndrome, VEGF

## To the Editor,

I read with interest the article by Vinod et al. [1] entitled “Kasabach-Merritt Syndrome in an Adult” in a recent issue of the Turkish Journal of Hematology.

Related to this article, I would like to add that Özsoylu [2] reported megadose methylprednisolone (MDMP) treatment for different hematological conditions including hemangiomas, infantile hemangiomatosis, and Kasabach-Merritt syndrome. Özsoylu [2] recommended the daily use of MDMP at 30 mg/kg for 3 days, then 20 mg/kg for 4 days and subsequently 10, 5, 2, and 1 mg/kg with each dose administered for 1 week around 6 AM as a single dose [2,3]. Özsoylu [2] treated patients who were resistant to conventional corticosteroid treatment either orally or intravenously over the course of 10-15 min [2,3]. Conventional corticosteroid treatment and MDMP were comparatively studied for the treatment of hemangiomas and it was observed that MDMP was superior [4].

In our department, eight patients with infantile hemangiomas were treated with interferon-a2a plus oral prednisolone without observing severe complications. In this study, the median age was 4 months (range: 3-12 months); six of them were female and two were male. Three cases were not followed and three patients achieved complete regression while a partial regression was observed in two others. The beneficial results were obtained with interferon-a2a plus oral prednisolone treatment in these cases [5].

On the other hand, vascular endothelial growth factor (VEGF) is recognized as an essential regulator of blood vessel growth. The use of anti-VEGF abolishes this vascular endothelial growth-promoting activity in vitro [6]. Three patients with periocular epithelioid hemangioma were treated with anti-VEGF successfully [7]. Although recent studies have indicated the effectiveness of anti-VEGF treatment in these cases, there is not a significant number of clinical trials reported.
