# Supplementary material for: Kasabach-Merritt Syndrome in an Adult: A Comment
Source: Turk J Haematol. 2019 Feb 7;36(1):52–3. doi: 10.4274/tjh.galenos.2018.2018.0356 (PMC6373517; doi:10.4274/tjh.galenos.2018.2018.0356)
Supplement: Related texts

# Reply to the Authors

**Authors:** Kolar Vishwanath Vinod, Joseph Johny, Mehalingam Vadivelan, Abdoul Hamide

## Dear Editor,

We would like to thank Gözdaşoğlu [1] for showing interest in our report of adult Kasabach-Merritt syndrome (KMS) [2]. Being adult hematologists, we should admit that we have limited experience with treatment of KMS, which by and large affects infants and young children. We do not have personal experience with interferon-alpha and have not studied the efficacy of different corticosteroids in different doses and combination of prednisolone with interferon-alfa for treatment of KMS. In this regard, we thank Gözdaşoğlu [1] for sharing her personal experience (effectiveness of prednisolone + interferon-alfa 2a combination therapy) with treatment of infantile hemangiomas and also for highlighting on reports of usefulness of high dose methylprednisolone [3] and anti-VEGF (bevacizumab) therapy for treatment of hemangiomas and KMS. Clinical trials evaluating different therapies would be desirable in the future to clarify on optimal treatment of KMS.

**Best Regards,**

## References

[1] Gözdaşoğlu S (2019). Kasabach-Merritt Syndrome in an Adult: A Comment. Turk J Hematol.

[2] Vinod KV, Johny J, Vadivelan M, Hamide A (2018). Kasabach-Merritt Syndrome in an adult. Turk J Hematol.

[3] Uysal KM, Olgun N, Erbay A, Sarıolioğlu F (2001). High-dose oral methylprednisolone therapy in childhood hemangiomas. Pediatr Hematol Oncol.
